# Identification of hepatoprotective traditional Chinese medicines based on the structure–activity relationship, molecular network, and machine learning techniques

**DOI:** 10.3389/fphar.2022.969979

**Published:** 2022-08-29

**Authors:** Shuaibing He, Yanfeng Yi, Diandong Hou, Xuyan Fu, Juan Zhang, Xiaochen Ru, Jinlu Xie, Juan Wang

**Affiliations:** ^1^ Key Laboratory of Vector Biology and Pathogen Control of Zhejiang Province, School of Medicine, Huzhou University, Huzhou Central Hospital, Huzhou, China; ^2^ Department of Life Sciences and Health, School of Science and Engineering, Huzhou College, Huzhou, China; ^3^ XinJiang Institute of Chinese Materia Medica and Ethnodrug, Urumqi, China; ^4^ School of Traditional Chinese Medicine, Zhejiang Pharmaceutical University, Ningbo, China

**Keywords:** machine learning, drug discovery, traditional Chinese medicine, predictive model, molecular network, hepatoprotection, structure–activity relationship

## Abstract

The efforts focused on discovering potential hepatoprotective drugs are critical for relieving the burdens caused by liver diseases. Traditional Chinese medicine (TCM) is an important resource for discovering hepatoprotective agents. Currently, there are hundreds of hepatoprotective products derived from TCM available in the literature, providing crucial clues to discover novel potential hepatoprotectants from TCMs based on predictive research. In the current study, a large-scale dataset focused on TCM-induced hepatoprotection was established, including 676 hepatoprotective ingredients and 205 hepatoprotective TCMs. Then, a comprehensive analysis based on the structure–activity relationship, molecular network, and machine learning techniques was performed at molecular and holistic TCM levels, respectively. As a result, we developed an *in silico* model for predicting the hepatoprotective activity of ingredients derived from TCMs, in which the accuracy exceeded 85%. In addition, we originally proposed a material basis and a drug property-based approach to identify potential hepatoprotective TCMs. Consequently, a total of 12 TCMs were predicted to hold potential hepatoprotective activity, nine of which have been proven to be beneficial to the liver in previous publications. The high rate of consistency between our predictive results and the literature reports demonstrated that our methods were technically sound and reliable. In summary, systematical predictive research focused on the hepatoprotection of TCM was conducted in this work, which would not only assist screening of potential hepatoprotectants from TCMs but also provide a novel research mode for discovering the potential activities of TCMs.

## 1 Introduction

As the largest solid organ in the human body, the liver is involved in the regulation of various important physiological processes, including metabolism, drug detoxification, glycogen storage, and bile secretion. In addition, the liver is also considered a major organ that protects against bacterial infection and foreign macromolecule invasion (Bedi et al., 2016). Therefore, dysfunction of the liver will lead to various liver diseases, including hepatitis, hepatic carcinoma, and nonalcoholic fatty liver. It was reported that the liver disease spectrum involved more than 103 liver diseases (Zhao et al., 2008). For hundreds of years, continuous efforts focused on preventing liver dysfunction have been made by scientists and hepatologists while liver diseases are still the major health burdens around the world (Wong et al., 2019). Moreover, recently, the spectrum of liver injury has been constantly changing, increasing the demand for developing novel liver-protecting agents (Huang et al., 2017). In light of these considerations, it is urgent and necessary to expand the spectrum of hepatoprotective drugs. Therefore, efforts focused on discovering potential hepatoprotective drugs are critical for relieving the burdens caused by liver diseases.

As an important branch of complementary and alternative medicine, TCM has made a non-negligible contribution to the development and continuation of human civilization in history. In China, TCM has been used to treat various liver diseases for centuries. In clinical application, lots of TCM products were discovered to have a good hepatoprotective effect. A classic example is silibinin, a flavonolignan derived from Silybi Fructus. Both *in vivo* and *in vitro* research practices have shown that silibinin protects liver cells against toxins (Saxena et al., 2022; Song et al., 2022). In clinical trials, silibinin was also used as a supplement to manage some chronic liver diseases (Ferenci et al., 2008). In addition, many other TCM products also exhibited significant liver-protecting effects, including glycyrrhizin, saikosaponin C, curcumin, dioscin, Lycii Fructus, Coptidis Rhizoma, and Notoginseng Radix Et Rhizome (Hong et al., 2015; Lam et al., 2016). All the studies mentioned earlier indicated that TCM is an essential resource for discovering hepatoprotective drugs.

Since it came into the 21st century, vast changes have taken place in the area of medical research. The rapid development of computing science and the continuous accumulation of biomedical data promote the change of the drug research mode from descriptive research to predictive research. Predictive research has the advantages of saving time, saving labor, and low cost. Furthermore, predictive research dramatically reduces the use of experimental animals in the process of drug research, which is in line with the basic idea of the animal welfare law to minimize the number of animals used in experiments (Moroy et al., 2012). Recently, predictive research has received continuously increasing attention. A series of predictive research studies have been carried out, which significantly promoted the process of drug development and discovery (Medina-Franco and Saldívar-González, 2020; Sessions et al., 2020). In summary, predictive research has become a vital and effective assistant strategy to discover novel drugs.

In history, TCM was primarily used in China and some other Asian countries. Recently, it has been started to be accepted and consumed by many western countries due to its unique effects in treating and preventing some intractable diseases (Martins, 2013; Wu et al., 2015). The widespread use of TCM products around the world encourages researchers to screen novel drugs from TCMs. In particular, with the successful development of artemisinin, expectations are higher to discover novel drugs with high efficacy and little harm from TCMs (Kong and Tan, 2015). Therefore, we believe that comprehensive predictive research focused on TCM-induced hepatoprotection is significant for discovering potential hepatoprotective drugs.

**FIGURE 1:**
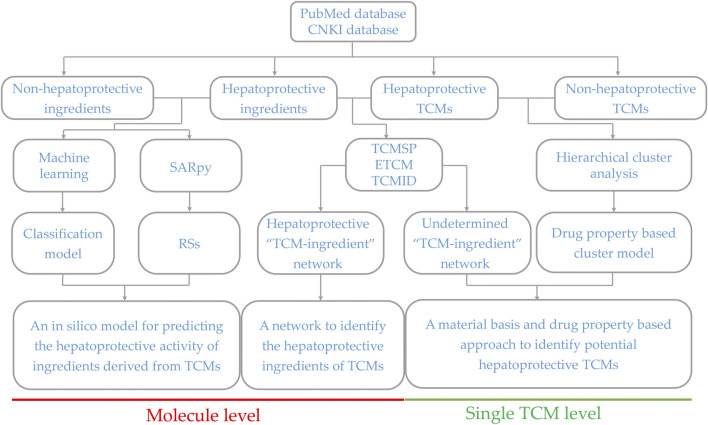
Schematic diagram of the systematic strategy for identifying hepatoprotective TCMs based on the structure–activity relationship, molecular network, and machine learning techniques. RSs, representative substructures.

Generally, sufficient data accumulation is necessary for conducting predictive research. Over the past decades, hundreds of TCM products have been reported to be beneficial to the liver, laying a solid foundation for discovering novel potential hepatoprotectants from TCMs *via* predictive research. Therefore, in the current study, systematical predictive research focused on TCM-induced hepatoprotection was conducted based on the hepatoprotective TCMs available in the literature at both molecular and complete singular TCM levels, through which we attempted to provide some valuable clues for discovering novel hepatoprotective agents from TCMs. The detailed experimental design is illustrated in Figure 1.

## 2 Results

### 2.1 Identification of the hepatoprotective activity of ingredients derived from traditional Chinese medicines based on the structure–activity relationship and machine learning

#### 2.1.1 *In silico* model for predicting the hepatoprotective activity of ingredients derived from traditional Chinese medicines

As described in Section 4.1, we established a large-scale dataset for TCM-induced hepatoprotection, including 538 hepatoprotective ingredients and 171 non-hepatoprotective ingredients. Then, seven machine learning algorithms were implemented within 5-fold cross-validation to develop hepatoprotective predictive models. As a result, a total of seven models were attained. As presented in Table 1, ACC (accuracy) of the models varied between 0.722 and 0.872, SE (sensitivity) varied between 0.712 and 0.968, SP (specificity) ranged from 0.567 to 0.754, and the AUC (the area under the receiver-operating characteristic curve) ranged from 0.712 to 0.884.

**TABLE 1:** Predictive power of the models developed based on eight machine learning algorithms.

Algorithm	Parameter	ACC	SE	SP	AUC
Naive Bayes	Default	0.722	0.712	0.754	0.816
J48	C = 0.45	0.825	0.898	0.596	0.712
K-star	B = 41	0.848	0.887	0.725	0.882
IBK	K = 1	0.843	0.877	0.737	0.807
Random forest	Depth = 0	0.872	0.968	0.567	0.884
Bagging	K = 1	0.841	0.879	0.719	0.854
AdaBoost	C = 0.05	0.853	0.922	0.637	0.859
Voting	—	0.865	0.913	0.713	0.890

The most accurate model was generated by the random forest algorithm with an ACC of 0.872. The random forest algorithm also produced the maximum SE (0.968) and AUC (0.884) values. Unfortunately, the SP (0.569) of the random forest model was very poor, which significantly decreased its practical application value. The most satisfactory SP (0.754) was provided by the Naive Bayes algorithm. However, ACC of the Naive Bayes model was only 72.2%, which was significantly lower than that of the other six models. The performance of the IBK model was similar to that of the KSTAR model, whereas the latter’s AUC was higher than the former’s by 7.5%. Therefore, in terms of comprehensive performance, the model generated by the K-star algorithm seems to be more satisfactory than the others.

In fact, we also attempted to integrate the advantages of the seven algorithms mentioned earlier *via* voting, aiming to improve the performance of the predictive model. Fortunately, almost all of the indicators of the voting model were higher than those of the KSTAR model slightly both for the training set and for the test set (Table 1 and Table 2). To test the predictive power of the voting model, an external validation set consisting of 135 hepatoprotective ingredients and 43 non-hepatoprotective ingredients was used. Consequently, the model’s ACC, SE, SP, and AUC were 0.871, 0.867, 0.884, and 0.953, respectively, indicating that our model was reasonably successful (Table 2). In summary, an *in silico* model for predicting the hepatoprotective activity of ingredients derived from TCMs was constructed for the first time. Both internal and external validation indicated that the model exhibited satisfactory predicting power. All of the predictive models generated in this work are available in Supplementary File S1.

**TABLE 2:** Comparison between the KSTAR model and the voting model on the test set.

Algorithm	ACC	SE	SP	AUC
K-star	0.848	0.837	0.884	0.948
Voting	0.871	0.867	0.884	0.953

#### 2.1.2 Representative substructures for the hepatoprotective activity

To understand the structural preference of the hepatoprotective ingredients, SARpy software was used to extract representative substructures (RSs) for hepatoprotection. As shown in Table 3, a total of 24 RSs were identified. Likelihood ratios (LRs) of the top 19 RSs were infinity, indicating that these substructures were only detected in hepatoprotectants. For the other five RSs, LRs ranged from 10.37 to 13.08, demonstrating that the occurrence probabilities of these substructures in hepatoprotectants were 10 times higher than those in non-hepatoprotectants. Considering the high bias of these substructures in hepatoprotectants, we could claim that these RSs mentioned earlier may be highly correlated with liver protection. Therefore, we recommend these RSs be taken into consideration in the design and modification of hepatoprotective drugs.

**TABLE 3:** Hepatoprotective RSs and their occurrences.

ID	RS	LR	Hepatoprotection/non-hepatoprotection (percentage)	Distribution of RSs
1	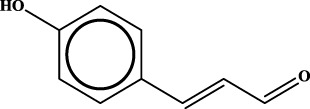	inf	45/0 (100.00%)	General phenylpropanoids (phenylpropionic acids (11); chalcones (4); lignans (3); simple coumarins (2)); phenylethanoid glycosides (9)
2	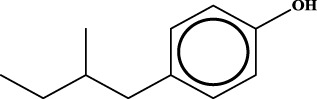	inf	44/0 (100.00%)	Lignans (34); flavanones/flavanonols (5)
3	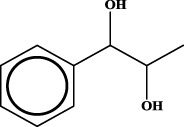	inf	39/0 (100.00%)	Flavonoids (25); lignans (dibenzocyclooctadienes (3); tetrahydrofurans (2)); coumarins (6)
4	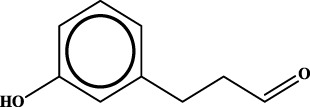	inf	30/0 (100.00%)	Flavanones/flavanonols (10); lignans (6); phenylpropionic acids (2); alkaloids (4)
5	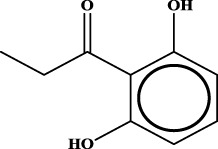	inf	28/0 (100.00%)	Flavanones/flavanonols (20); dihydrochalcones (4); xanthones (3)
6	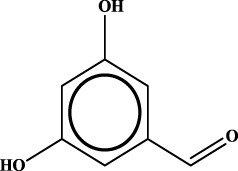	inf	25/0 (100.00%)	Tannins (6); flavonoids (6); anthraquinones (6)
7	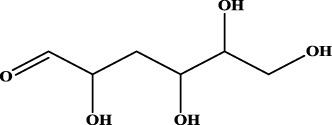	inf	24/0 (100.00%)	Flavones/flavonols (5); phenylpropionic acids (4); organic acids (4); oleanane-type triterpenoids (5)
8	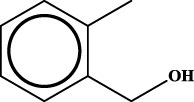	inf	16/0 (100.00%)	Lignans (5); quinonoids (3)
9	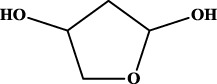	inf	16/0 (100.00%)	Terpenoids (6); steroidal saponins (3); oligosaccharides (3); flavonoids (3)
10	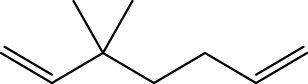	inf	15/0 (100.00%)	Terpenoids (oleanane-type triterpenoids (5); sesquiterpenes (5); others (5))
11	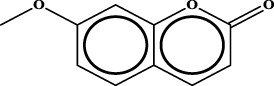	inf	15/0 (100.00%)	Coumarins (simple coumarins (7); pyranocoumarins (5); furanocoumarins (2); others (1))
12	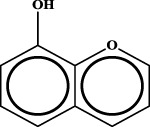	inf	14/0 (100.00%)	Flavones/flavonols (5); xanthones (2); furanocoumarins (3); simple coumarins (2)
13	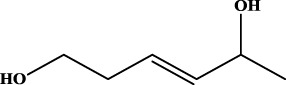	inf	14/0 (100.00%)	Terpenoids (cucurbitane triterpenoids (4); iridoids (4); others (4))
14	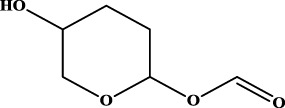	inf	14/0 (100.00%)	Terpenoids (oleanane-type triterpenoids (4); others (2)); simple coumarins (3)
15	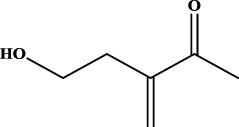	inf	12/0 (100.00%)	Terpenoids (kauran diterpenes (3); bicyclic diterpenoids (3); others (1))
16	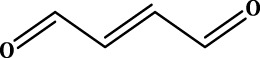	inf	12/0 (100.00%)	Quinonoids (naphthoquinones (3); p-Benzoquinones (3))
17	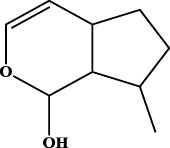	inf	10/0 (100.00%)	Iridoids (10)
18	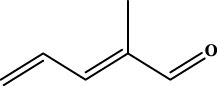	inf	10/0 (100.00%)	Terpenoids (5); alkaloids (3)
19	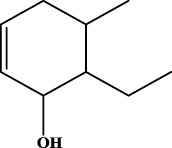	inf	10/0 (100.00%)	Terpenoids (oleanane-type triterpenoids (4); others (2))
20	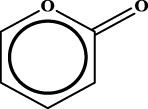	13.08	41/1 (97.62%)	Coumarins (pyranocoumarins (5); furanocoumarins (11); simple coumarins (15); others (3)); cardiac glycosides (4)
21	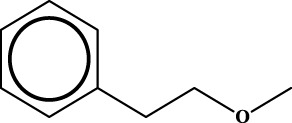	12.44	39/1 (97.50%)	Phenylethanoid glycosides (10); flavonoids (9); coumarins (pyranocoumarins (4); others (1))
22	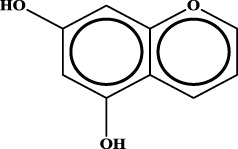	12.28	77/2 (97.47%)	Flavonoids (flavones/flavonols (61); others (14))
23	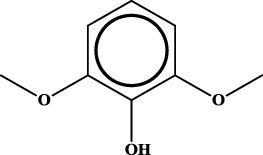	10.53	33/1 (97.06%)	Lignans (dibenzocyclooctadienes (11); arylnaphthalenes (4); biphenylenes (3); others (3)); bibenzyles (4)
24	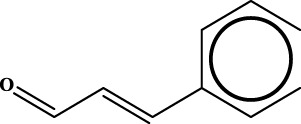	10.37	65/2 (97.01%)	Phenylpropanoids (phenylpropionic acids (12); others (2)); phenylethanoid glycosides (9); flavonoids (chalcones (7); others (3)); amide alkaloids (5); terpenoids (8)

Moreover, the distribution of the RSs was also explored (Table 3). As a result, we found that a total of 10 RSs [lignans (ID2 and ID23), flavonoids (ID3, ID5, and ID21), terpenoids (ID9, ID13, and ID17), and coumarins (ID11 and ID20)] were largely distributed into specific compound families. In contrast, the other 14 RSs showed lower specificity to the structural category by associating with multiple compound families. It has been a consensus that compounds with similar structures tend to hold consistent activities. Therefore, more attention should be paid to the compound families containing frequent hepatoprotective ingredients when screening hepatoprotective drugs from TCMs.

### 2.2 Comprehensive analysis focused on the hepatoprotective activity of traditional Chinese medicine in the singular traditional Chinese medicine level based on bioinformatics

#### 2.2.1 Efficacy category analysis

**FIGURE 2:**
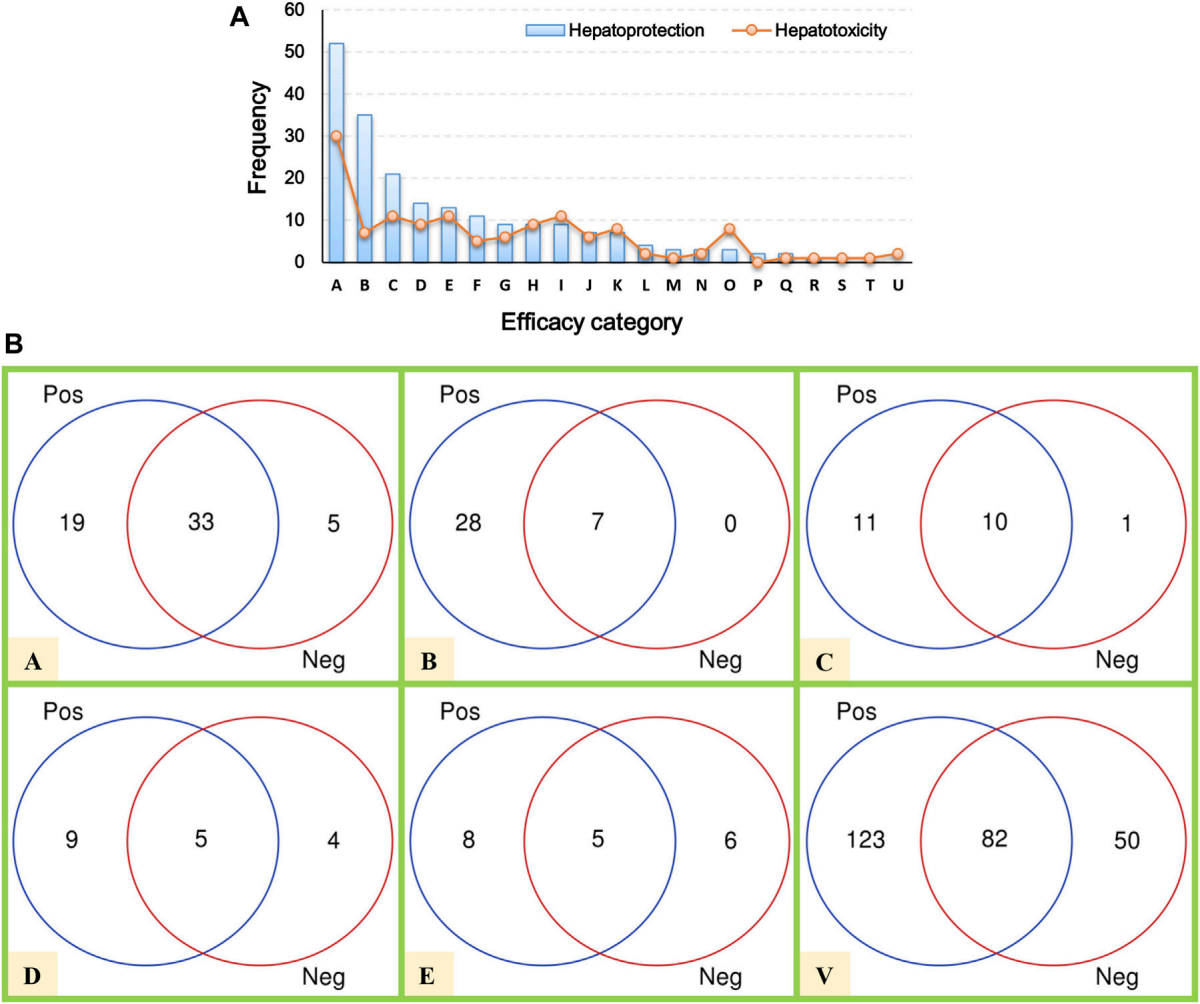
Efficacy category analysis. **(A)** Efficacy category of the hepatoprotective and hepatotoxic TCMs. **(B)** Overlap between hepatoprotective and hepatotoxic TCMs.

Here, a total of 205 hepatoprotective TCMs were collected (Supplementary File S2). Despite a systematical literature retrieval being conducted, we must acknowledge that there still existed some hepatoprotective TCMs that escaped from our vision. Therefore, TCMs with the liver-protecting activity included but not limited to those 205 TCMs mentioned earlier. To answer the question of which efficacy categories are rich in hepatoprotective TCMs, a systematical efficacy category analysis was conducted. The results showed that the hepatoprotective TCMs collected in this work were categorized into 18 types. As illustrated in Figure 2A, the antipyretics rank first with a frequency of 52, followed by the tonifying medicinal (35), blood-activating stasis-removing drugs (21), diaphoretics (14), expectorant antitussive antiasthmetics (13), and qi-regulating drugs (11). For the other 12 types of TCMs, the frequencies of hepatoprotective TCMs were less than 10.

In our previous study, a hepatotoxic herb list consisting of 132 members was developed, making it possible to conduct a comparative analysis between hepatoprotective and hepatotoxic TCMs (He et al., 2019b). As shown in Figure 2A, the TCMs with liver toxicity were divided into 21 types. The antipyretics were the leading cause of herb-induced liver injury with a count of 30. The wind damp-dispelling drugs (11), expectorant antitussive antiasthmetics (11), blood-activating stasis-removing drugs (11), diuretic dampness excreting drugs (9), and diaphoretics (9) rank second, third, fourth, fifth, and sixth, respectively. Further comparative analysis revealed that there existed plenty of overlap between the hepatoprotective and hepatotoxic TCMs (Figure 2B). To be exact, a total of 82 hepatoprotective TCMs were reported to be implicated by liver injury to a different extent, accounting for 40 percent of the hepatoprotective TCMs collected in this work. The phenomenon mentioned earlier indicates that some hepatoprotective TCMs may exert an adverse effect on liver. Therefore, physicians and hepatologists should keep an eye on hepatoprotectant-induced liver injury in clinical settings.

In summary, the antipyretics, tonifying medicinal, and blood-activating stasis-removing drugs were the three main sources of hepatoprotective TCMs. However, both of the antipyretics and blood-activating stasis-removing drugs were also the most implicated agents of herb-induced liver injury simultaneously. In contrast, as the second major efficacy category of hepatoprotective TCMs, the tonifying medicinal was rarely reported to induce liver toxicity. Therefore, we speculated that tonifying medicinals may be an important and safe source for discovering novel hepatoprotective TCMs.

#### 2.2.2 Drug property analysis

**FIGURE 3:**
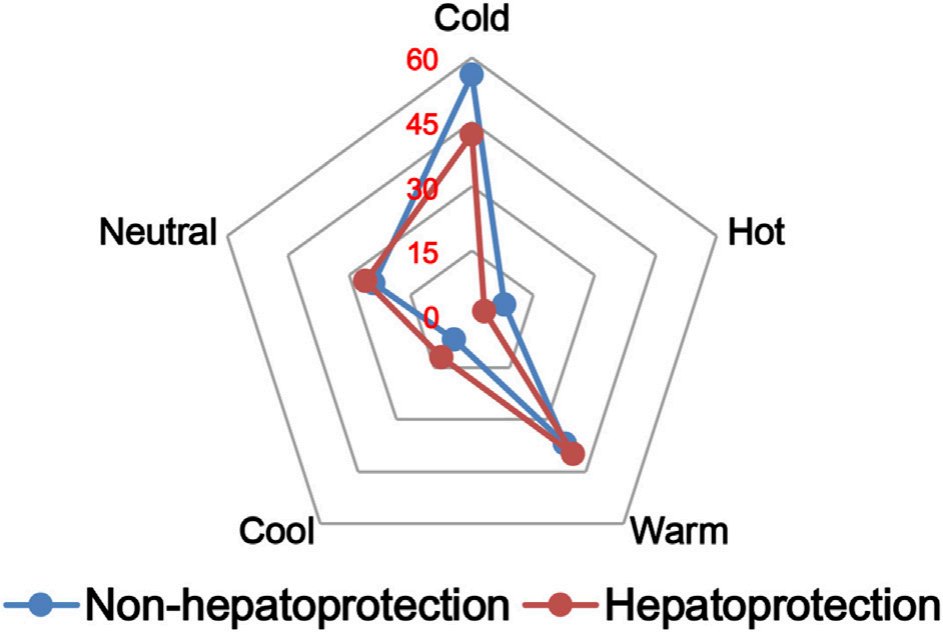
Frequency distribution of hepatoprotective and non-hepatoprotective TCMs in four properties.

**FIGURE 4:**
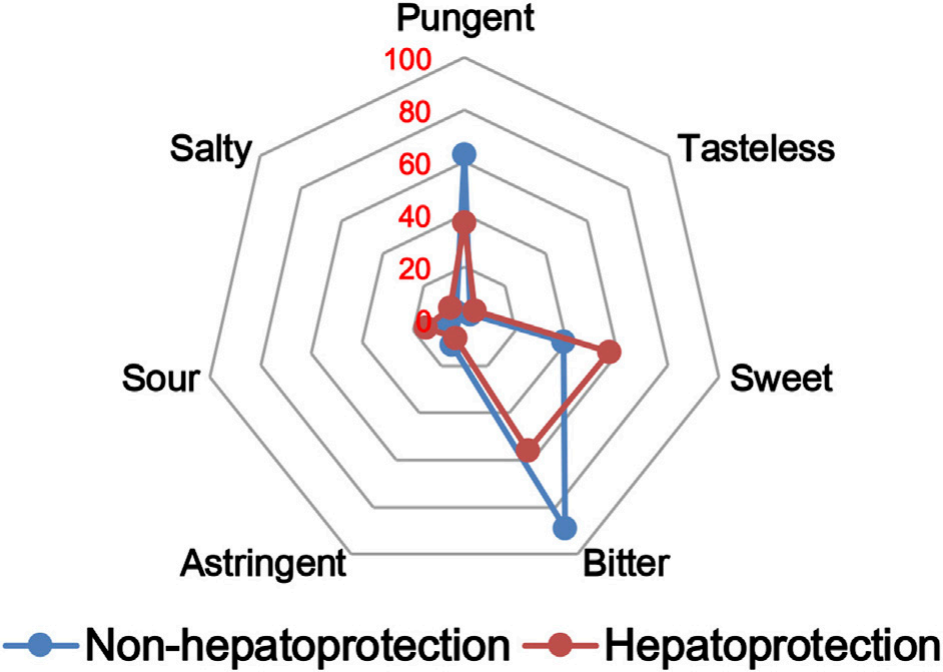
Frequency distribution of hepatoprotective and non-hepatoprotective TCMs in five flavors.

**FIGURE 5:**
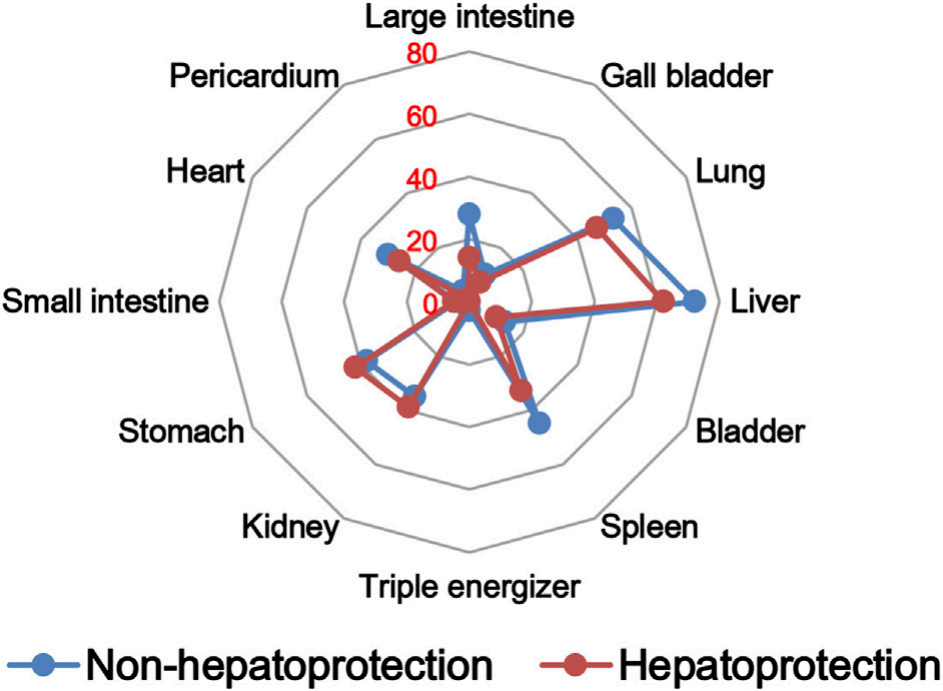
Frequency distribution of hepatoprotective and non-hepatoprotective TCMs in channel tropism.

The TCM theory considered that the drug property is responsible for specific efficacy and toxicity of TCMs (Ung et al., 2007; Chen et al., 2018). Previous studies have also reported that there exists a certain degree of correlation between drug properties and hepatotoxicity (Liu et al., 2016). To answer the question of whether drug properties are related to the generation of hepatoprotection or not, drug properties of the hepatoprotective TCMs were investigated, including four properties, five flavors, and channel tropism. As there was rarely any literature focusing on the topic of non-hepatoprotection of TCMs, it was difficult to collect non-hepatoprotective TCMs. Therefore, in this study, TCMs with any potential liver toxicity were defined as non-hepatoprotective TCMs. Only those TCMs beneficial to the liver and without liver adverse effects were regarded as hepatoprotective TCMs. As a result of this, a total of 123 hepatoprotective TCMs and 132 non-hepatoprotective TCMs were attained and used to conduct drug property analysis (Supplementary File S3). We found that compared to non-hepatoprotective TCMs, hepatoprotective TCMs were inclined to show sour or sweet flavor, whereas less likely to show cold property, bitter flavor, pungent flavor, large intestine channel, and spleen channel slightly (Figures 3, 4, 5). In fact, pungent flavor was reported to be a risk factor for hepatotoxicity in a previous study (Liu et al., 2016), which was consistent with the phenomenon observed in the present study.

To further quantify the difference in drug properties between hepatoprotective and non-hepatoprotective TCMs, the chi-squared test was conducted. The results indicated that drug properties of the hepatoprotective TCMs were significantly different from those of the non-hepatoprotective TCMs (*p* < 0.05). We must acknowledge that the aforementioned *p*-value attained is close to 0.05 (*p* = 0.042). This phenomenon may be due to those hepatoprotective TCMs implicated in herb-induced liver injury being defined as non-hepatoprotective TCMs during the comparative analysis, which decreased the drug property difference between hepatoprotective TCMs and non-hepatoprotective TCMs to a certain extent. In fact, among those 132 non-hepatoprotective TCMs, a total of 82 TCMs showed biphasic effects on the liver, including hepatoprotection and hepatotoxicity. Nevertheless, there indeed exists significant difference of the drug property between hepatoprotective TCMs and non-hepatoprotective TCMs. Therefore, we can conclude that drug property is related to the generation of hepatoprotection. TCMs with similar drug properties to the hepatoprotective TCMs may exhibit a potential liver-protecting activity.

#### 2.2.3 Association rules analysis to explore the relationship between the drug property and hepatoprotective activity

In Section 2.2.2, we found that there existed a certain degree of correlation between the drug property and hepatoprotection. To further reveal the detailed association relationship, association rules analysis was conducted. Consequently, a total of five association rules were attained. There was one rule with a single item, three rules with double items, and one rule with triple items (Table 4). The rule with the ID of 3 demonstrated that sour flavor was significantly related to hepatoprotection, which consisted of the TCM theory of “sour into liver” (Wang and Jing, 2019). In addition, sweet flavor showed strong association with hepatoprotection by incorporating with the kidney channel, warm property, or stomach channel, respectively. As the only rule that consisted of triple items, the rule with an ID of 4 involved sweet flavor, liver channel, and kidney channel. The association rules attained previously revealed the relationship between the drug property and hepatoprotection preliminarily. Theoretically, TCM with these drug properties may have a high potential to generate beneficial effects on the liver.

**TABLE 4:** Results of association rules analysis (support ≥5%, confidence ≥65%, lift >1).

ID	Rule	Support (%)	Confidence (%)	Lift
1	{sweet, kidney}⇒{hepatoprotection}	10.59	69.23	1.44
2	{sweet, warm}⇒{hepatoprotection}	6.67	73.91	1.53
3	{sour}⇒{hepatoprotection}	5.88	71.43	1.48
4	{sweet, liver, kidney}⇒{hepatoprotection}	5.49	66.67	1.38
5	{sweet, stomach}⇒{hepatoprotection}	6.67	65.38	1.36

#### 2.2.4 “Traditional Chinese medicine-ingredient” network focused on hepatoprotection

**FIGURE 6:**
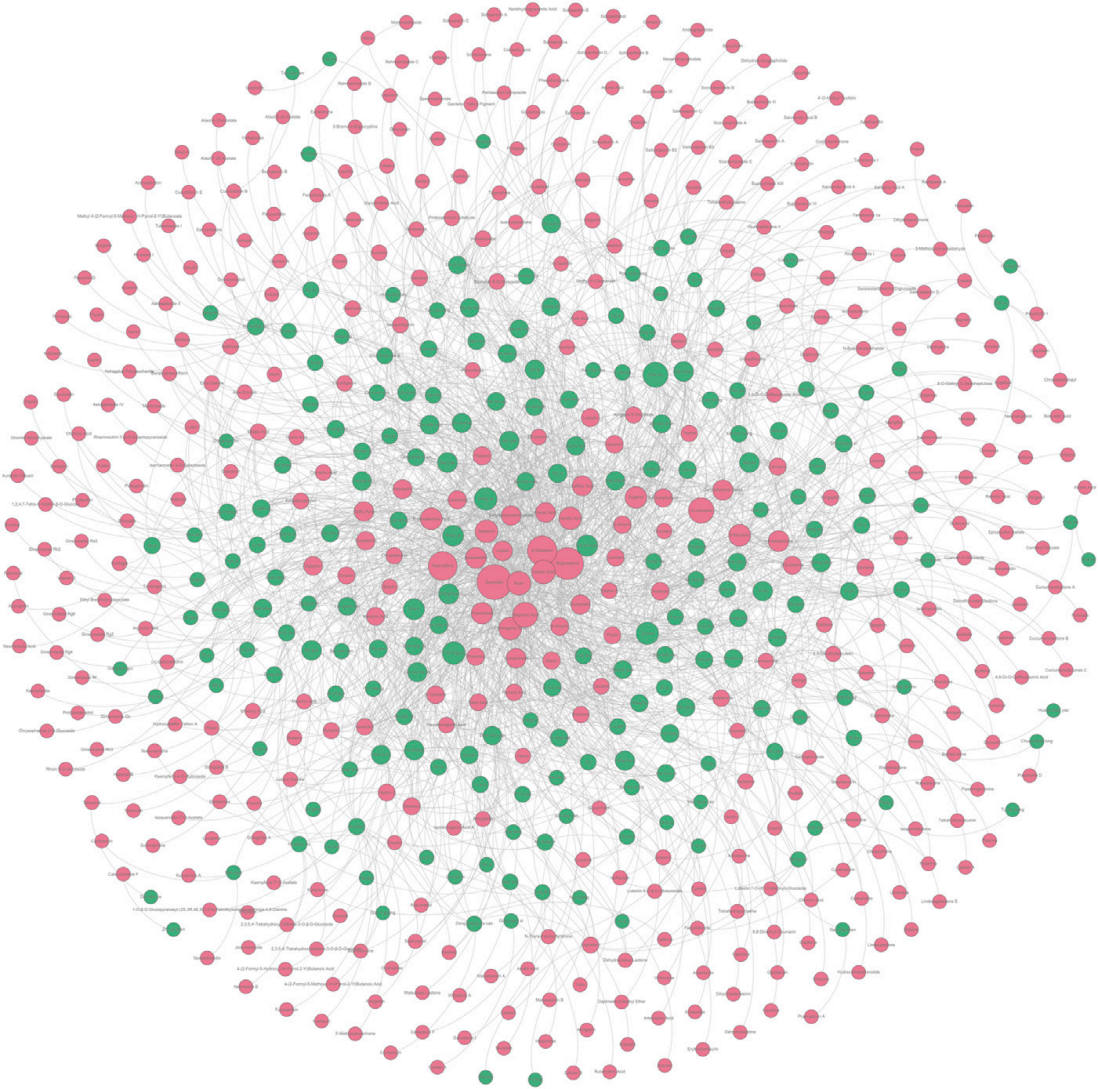
“TCM-ingredient” network focused on hepatoprotection. The hepatoprotective TCMs and the hepatoprotective ingredients were displayed by green and red nodes, respectively. If a TCM and an ingredient were connected by a gray line, it indicated that the TCM contained the ingredient.

In Section 2.1.1 and Section 2.2.1, we collected a series of potential hepatoprotective ingredients and TCMs. Theoretically, there should exist some associative relationships between these ingredients and TCMs. To discover the detailed relationships, we constructed a “TCM-ingredient” network (Figure 6, Supplementary File S4). The network consisted of 638 nodes and 2262 edges. A total of 205 TCMs and 433 compounds were involved. This network intuitively displayed the association relationships between the hepatoprotective TCMs and ingredients, which may help researchers identify the hepatoprotective ingredients of specific TCMs.

**FIGURE 7:**
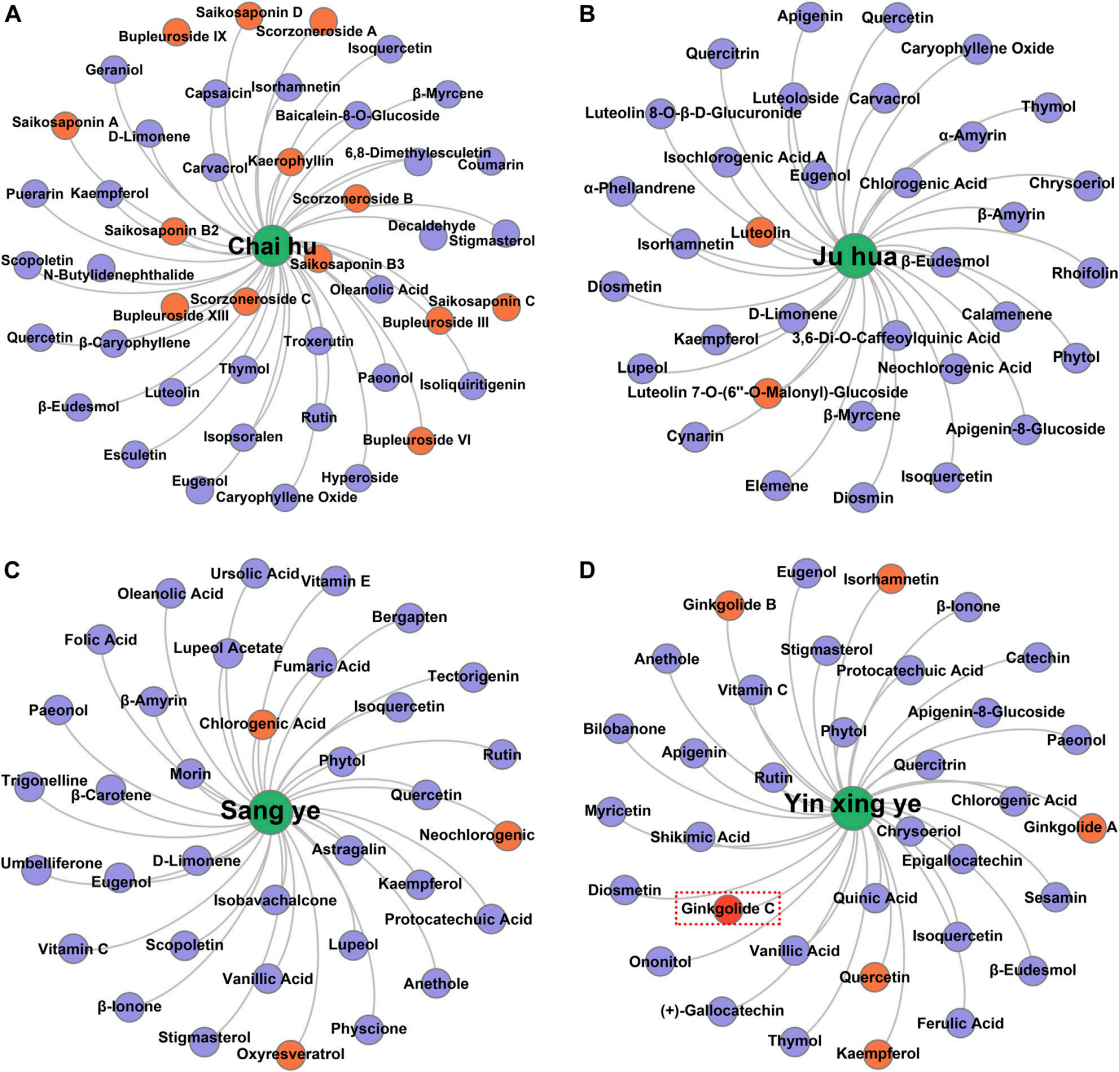
Identification of the hepatoprotective ingredients in Chai hu **(A)**, Ju hua **(B)**, Sang ye **(C)**, and Yin xing ye **(D)** based on the hepatoprotective “TCM-ingredient” network.

Taking several commonly used TCMs as cases, we identified the hepatoprotective ingredients in Chai Hu (A), Ju Hua (B), Sang Ye (C), and Yin Xing Ye (D) based on the hepatoprotective “TCM-ingredient” network. As demonstrated in Figure 7, only 13 ingredients were identified to be associated with the hepatoprotective effect of Chai Hu *via* direct literature retrieval (Ashour and Wink, 2011; Lee et al., 2012; Qi et al., 2013; Lin et al., 2015). Interestingly, the hepatoprotective “TCM-ingredient” network discovered 44 hepatoprotective ingredients for Chai Hu. In fact, systematical literature retrieval only led to 2 (Sugawara and Igarashi, 2009), 3 (Lee et al., 2017; Lee et al., 2018), and 6 (Wei et al., 2014; Yang M. H. et al., 2020; Yang Y. et al., 2020; Sarkar et al., 2020) hepatoprotective ingredients for Ju Hua, Sang Ye, and Yin Xing Ye. In contrast, the “TCM-ingredient” network discovered 34, 35, and 34 hepatoprotective ingredients for the corresponding TCMs, respectively. The results mentioned earlier indicated that the hepatoprotective “TCM-ingredient” network was able to identify the hepatoprotective ingredients of TCMs in a more comprehensive and effective way than direct literature retrieval.

Of note, ginkgolide C, a hepatoprotective ingredient derived from Yin Xing Ye, was not identified successfully by the hepatoprotective “TCM-ingredient” network. Literature searches found that a total of two studies reported the hepatoprotective activity of ginkgolide C (Huang W. C. et al., 2018; Yang M. H. et al., 2020). However, there was no TCM-related vocabulary that existed in the abstracts or titles of these two studies. Therefore, ginkgolide C escaped our vision when we collected hepatoprotective ingredients and was not included in the hepatoprotective “TCM-ingredient” network. In the future, with the introduction of more hepatoprotective ingredients to the hepatoprotective “TCM-ingredient” network, the network will produce a more satisfactory performance.

### 2.3 Material basis and drug property–based approach to identify potential hepatoprotective traditional Chinese medicines

Theoretically, TCMs may generate a certain degree of liver-protecting effect when they satisfy the following two conditions: they contain rich hepatoprotective ingredients and possess similar drug properties to the hepatoprotective TCMs. Therefore, in this section, first, we identified TCMs containing rich hepatoprotective ingredients by constructing an undetermined “TCM-ingredient” network. Then, a comparison between the TCMs containing rich hepatoprotective ingredients and the hepatoprotective TCMs was conducted through cluster analysis. Finally, the TCMs with drug properties similar to those of the hepatoprotective TCMs were considered to be potential hepatoprotective TCMs.

#### 2.3.1 Identification of traditional Chinese medicines containing rich liver-protecting ingredients

**FIGURE 8:**
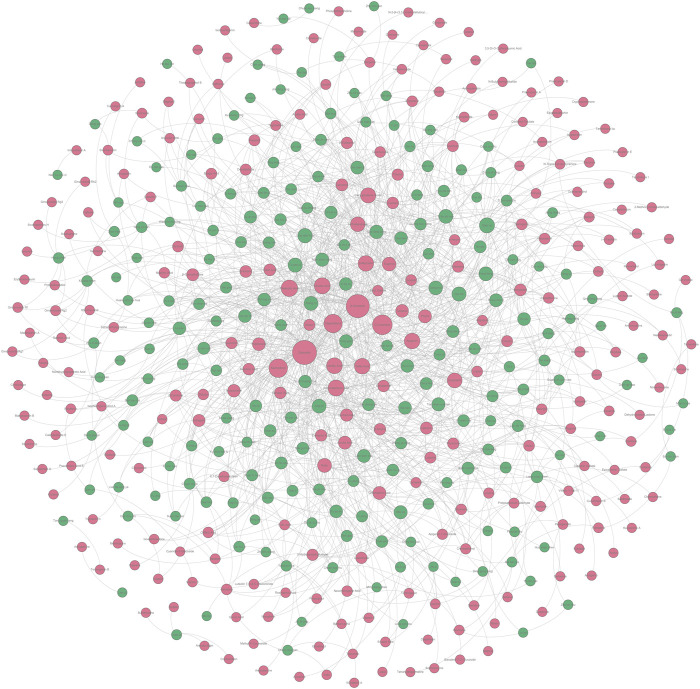
Undetermined “TCM-ingredient” network. Green and red nodes represented the TCMs and the hepatoprotective ingredients, respectively. The gray lines connecting the nodes indicated that the TCMs contain the ingredients.

According to the method mentioned in Section 4.3.4, we constructed an undetermined “TCM-ingredient” network by integrating TCMs and hepatoprotective ingredients. Of note, those 205 hepatoprotective TCMs mentioned in Section 2.2.1 were removed from this network. In other words, for any one of the TCMs included in this network, whether or not it holds the hepatoprotective activity was unclear. As shown in Figure 8 (Supplementary File S5), the network consisted of 443 nodes and 1376 edges. A total of 197 TCMs and 246 hepatoprotective ingredients were involved. Degree analysis revealed that a total of 26 TCMs contained liver-protecting ingredients greater than 15 (Table 5). Theoretically, the more liver-protecting ingredients a TCM contains, the more likely it is to produce a liver-protecting effect. Therefore, these 26 TCMs were considered as candidate hepatoprotective TCMs.

**TABLE 5:** Top 26 TCMs containing rich liver-protecting ingredients.

ID	TCM	Number of hepatoprotective components	Association rules	Serial number
1	Qian Hu	25	—	U19
2	Ling Xiao Hua	22	1	U16
3	Fu Pen Zi	21	1, 2, 5	U5
4	Gui Zhi	21	4	U10
5	E Bu Shi Cao	20	—	U3
6	Fang Feng	20	4	U4
7	Gao Ben	20	—	U7
8	Jing Jie	20	—	U13
9	Xiang Ru	20	—	U24
10	Qiang Huo	19	—	U20
11	Gao Liang Jiang	18	—	U6
12	Mai Ya	17	3	U17
13	Man Shan Hong	17	—	U18
14	Qing Guo	17	1, 3	U21
15	Tian Shan Xue Lian	17	—	—
16	Che Qian Zi	16	2, 5	U2
17	Hua Ju Hong	16	—	U12
18	Lian Qian Cao	16	—	U15
19	Bai Lian	15	—	U1
20	Gou Gu Ye	15	—	U8
21	Gu Sui Bu	15	—	U9
22	Hu Lu Ba	15	—	U11
23	La Jiao	15	—	U14
24	Wei Ling Cai	15	—	U22
25	Xi He Liu	15	3	U23
26	Zhi Shi	15	1	U25

**FIGURE 9:**
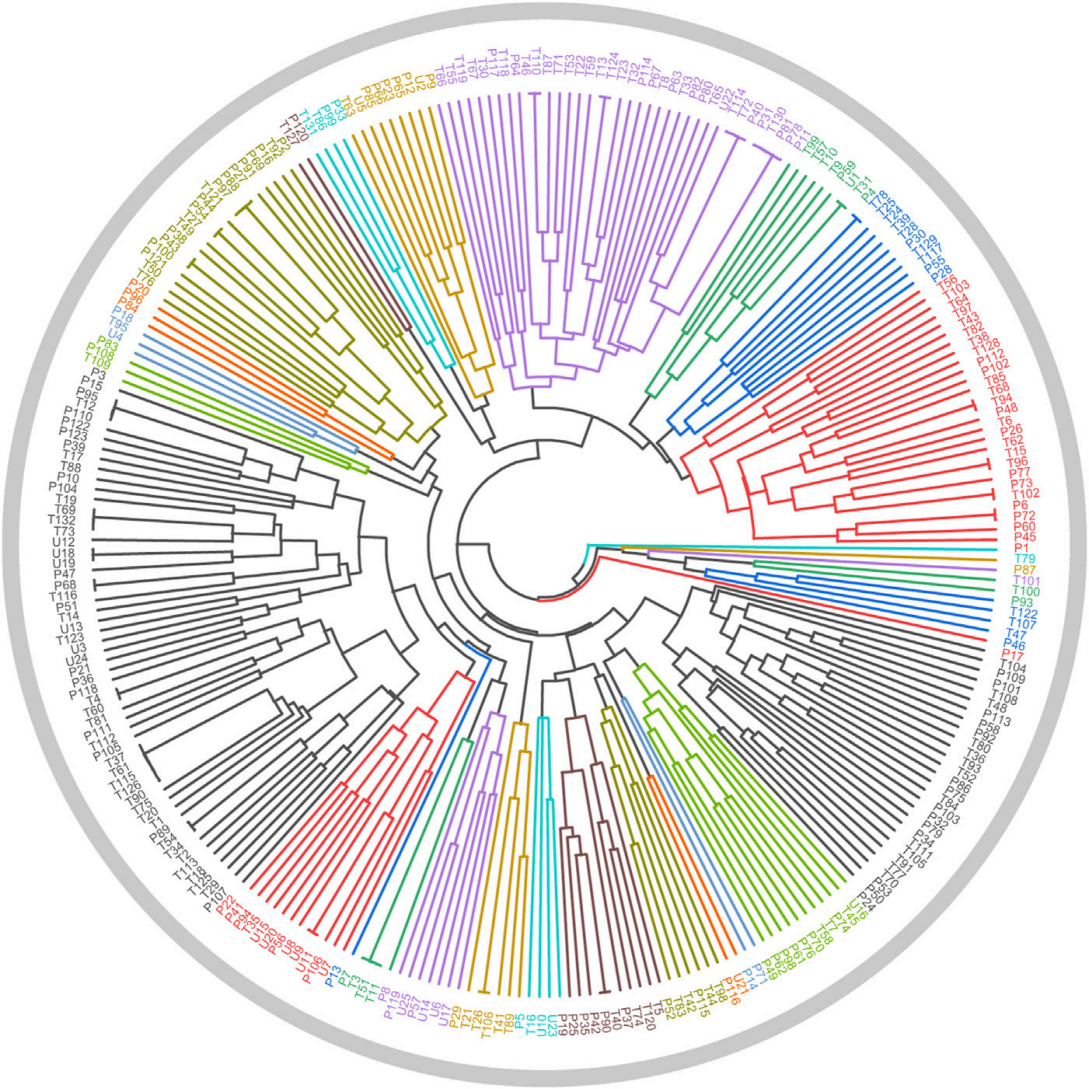
Cluster analysis based on drug property. The samples with the prefixes of P and T indicated the hepatoprotective TCMs and the non-hepatoprotective TCMs, respectively. U1–U25 represented the 25 samples to be tested.

Serial number corresponds to the TCM ID in Figure 9; ID in column of association rules corresponds to ID in Table 4.

#### 2.3.2 Identification of traditional Chinese medicines with drug properties similar to those of the hepatoprotective TCMs by cluster analysis

In Section 2.2.2, it has been demonstrated that the drug properties of the hepatoprotective TCMs are significantly different from those of the non-hepatoprotective TCMs. Herein, taking the drug properties of the hepatoprotective TCMs (123) and the non-hepatoprotective TCMs (132) as input (Supplementary File S3), hierarchical cluster analysis (Origin 9.0 software) based on Euclidean distance was conducted to develop a cluster model. This model was able to evaluate whether or not the drug properties of specific TCMs were closer to those of the hepatoprotective TCMs. For specific TCM, if it was clustered into the same branch with the hepatoprotective TCMs, its drug property was considered to be closer to the drug properties of the hepatoprotective TCMs. Otherwise, its drug properties were considered to be closer to the drug properties of the non-hepatoprotective TCMs.

In Section 2.3.1, a total of 26 TCMs were found to contain rich liver-protecting ingredients. Of note, Tian Shan Xue Lian lacked channel tropism information and could not be measured by our cluster model. Therefore, only 25 TCMs were put into the cluster model (Figure 9). As a result, a total of 12 TCMs, namely, Ling Xiao Hua, Fu Pen Zi, Gao Ben, Gao Liang Jiang, Mai Ya, Qing Guo, Che Qian Zi, Hu Lu Ba, Gou Gu Ye, Gu Sui Bu, Zhi Shi, and La Jiao, were discovered to hold similar drug properties to the hepatoprotective TCMs. In summary, these 12 TCMs contained rich liver-protecting ingredients and also held similar drug properties to the hepatoprotective TCMs. Therefore, we speculated that these 12 TCMs may have potential hepatoprotective activity. Detailed descriptions of these 12 TCMs are provided in Table 6.

**TABLE 6:** Twelve potential hepatoprotective TCMs.

ID	Name	Origin plants	Efficacy category	Hepatoprotective activity
1	Ling Xiao Hua	*Campsis grandiflora* (Thunb.) K. Schum. and *Campsis radicans* (L.) Seem	Blood-activating stasis-removing drugs	—
2	Fu Pen Zi	*Rubus chingii* Hu	Astringent medicinal	Ameliorates CCl₄-induced liver fibrosis Wu et al. (2022)
3	Gao Ben	*Ligusticum sinense* Oliv. and *Ligusticum jeholense* Nakai et Kitag	Diaphoretics	—
4	Gao Liang Jiang	*Alpinia officinarum* Hance	Warming interior drugs	Anti-hepatoma Zhang et al. (2012); Su et al. (2013); Abass et al. (2018); Fang et al. (2019); anti-hepatitis Luo et al. (2015); nonhepatotoxicity Li et al. (2018)
5	Mai Ya	*Hordeum vulgare* L.	Digestants	Alleviates alcohol-induced hepatocellular injury Park et al. (2021)
6	Qing Guo	*Canarium album* Raeusch	Antipyretics	Ameliorates hepatic lipid accumulation Yeh et al. (2018); ameliorates CCl₄/D-galactosamine-induced liver injury Tamai et al. (1989); Ito et al. (1990)
7	Che Qian Zi	*Plantago asiatica* L., *Plantago depressa* Willd	Diuretic dampness excreting drugs	Against lipopolysaccharide-induced liver injury Li et al. (2019)
8	Hu Lu Ba	*Trigonella foenum-graecum* L.	Tonifying medicinal	Alleviates chemical and drug-induced liver injury (alcohol, thioacetamide, cypermethrin, cadmium, bleomycin, thiamethoxam, adriamycin, AlCl_3_, and gasoline fumes) Kaviarasan et al. (2006); Kaviarasan and Anuradha (2007); Kaviarasan et al. (2007); Kaviarasan et al. (2008); Sushma and Devasena (2010); Lu et al. (2012); Sakr and Abo-El-Yazid (2012); Shivashankara et al. (2012); Belaïd-Nouira et al. (2013); Arafa et al. (2014); Zargar (2014); Qureshi et al. (2016); Kandhare et al. (2017); Abdrabouh (2019); Feki et al. (2019); Fatima Zaidi and Masood (2020); induces hepatoma cell apoptosis Khalil et al. (2015); improves non-alcoholic fatty liver Mohamed et al. (2015); nonhepatotoxicity Serrano (2014)
9	Gou Gu Ye	*Ilex cornuta* Lindi. ex Paxt	Antipyretics	Prevents high-fat diet-induced fatty liver Liu et al. (2022)
10	Gu Sui Bu	*Drynaria fortunei* (Kunze) J. Sm	Tonifying medicinal	—
11	Zhi Shi	*Citrus aurantium* L. and *Citrus sinensis* Osbeck	Qi-regulating drugs	Alleviates chemical and drug-induced liver injury (acetaminophen, methotrexate, alcohol, and CCl₄) Choi et al. (2015); Kim et al. (2016); Lim et al. (2016); Hsouna et al. (2018); He et al. (2019a); Ben Hsouna et al. (2019); Shu et al. (2020); prevents non-alcoholic fatty liver disease Han et al. (2019)
12	La Jiao	*Capsicum annuum* L	External medicinal (draw out toxin, resolve putridity)	Attenuates liver fibrosis Sheng et al. (2020); anti-hepatoma carcinoma cell proliferation Chu et al. (2002); induces hepatoma carcinoma cell apoptosis Huang et al. (2009); improves nonalcoholic fatty liver disease Joo et al. (2021); alleviates alcohol-induced liver injury Koneru et al. (2018)

#### 2.3.3 Twelve potential hepatoprotective traditional Chinese medicines

As demonstrated in Table 6, among these 12 potential hepatoprotective TCMs, a total of six TCMs (including Ling Xiao Hua, Gao Ben, Qing Guo, Hu Lu Ba, Gou Gu Ye, and Gu Sui Bu) belonged to the top four efficacy categories of the hepatoprotective TCMs. In other words, about 50% of the novel potential hepatoprotective TCMs were derived from the hepatoprotective TCM-productive efficacy categories, reflecting the reliability of our results to a certain extent.

In Section 2.2.3, we attained five rules which were highly correlated with hepatoprotection. Here, the occurrence frequencies of these five rules in those 25 TCMs were investigated. As a result, the frequency of these rules in these 12 potential hepatoprotective TCMs was 10. However, in the other 13 TCMs, the frequency of these rules was only 3. This phenomenon indicated that these 12 potential hepatoprotective TCMs were more likely to protect the liver than the other 13 TCMs in terms of drug property.

In fact, 9 out of these 12 TCMs were reported to produce beneficial effects to the liver in animal or cell experiments (Table 6). For Ling Xiao Hua and Gao Ben, although they were not reported to treat liver disorders, none of them was implicated by drug-induced liver injury. Direct evidence focused on the liver protection of Gu Sui Bu was unavailable. However, Li Ye Hu Jue (*Drynaria quercifolia* (L.) J. Sm), a form of TCM which has the same effect with Gu Sui Bu, has been demonstrated to exhibit protection against rat liver fibrosis induced by carbon tetrachloride through the Nrf2/ARE and NFκB signaling pathways (Anuja et al., 2018). The results provided by this work were highly consistent with the reports in previous publications and further confirmed the effectiveness of our methods.

## 3 Discussion

In this work, the first contribution is that a comprehensive structure–activity relationship study focused on the hepatoprotective activity of TCMs was conducted. Initially, an *in silico* model for predicting the liver protection of phytoconstituents was developed based on eight machine learning algorithms. Both the five-fold cross-validation and the external validation produced ACC values that exceeded 85%, indicating that the model exhibited satisfactory predicting power. Of note, the imbalance of the dataset may affect the model’s performance; a dataset with sufficient non-hepatoprotective phytoconstituents may improve our model’s predicting power. Nevertheless, we attempted to develop a model for predicting the hepatoprotection of ingredients derived from TCMs for the first time. This model would contribute to narrowing the scope of candidate drugs in the discovery of novel hepatoprotectants. Second, the structural preference of the hepatoprotective phytoconstituents was investigated. Consequently, a total of 24 RSs for hepatoprotection were identified. Theoretically, phytoconstituents containing these RSs are more likely to produce beneficial effects on the liver. Therefore, these RSs would provide valuable guidance for the design and structural modification of novel hepatoprotectants.

Except the efforts in exploring the hepatoprotection of TCMs in the molecular level, a comprehensive analysis at the level of holistic TCM was also conducted. A total of 205 hepatoprotective TCMs were collected. Efficacy category analysis showed that the top four efficacy categories were the antipyretics, the tonifying medicinals, the blood-activating stasis-removing drugs, and the diaphoretics, respectively. These four efficacy categories contained many hepatoprotective TCMs. In fact, 6 out of those 12 novel potential hepatoprotective TCMs identified in Section 2.3.2 belonged to the top four efficacy categories, further indicating that these four groups were important sources of the hepatoprotective TCMs. Then, focused on the drug property, a chi-squared test was performed. It showed that the drug properties of the hepatoprotective TCMs were significantly distinguished from those of the non-hepatoprotective TCMs, reflecting that there exists a certain degree of correlation between the drug property and liver-protecting activity. To investigate the detailed relationship, association rules analysis was performed. Subsequently, a total of five association rules were identified. These rules would help explain the liver protection of TCMs from the perspective of the drug property. In fact, more association rules could be attained by decreasing the thresholds of support and confidence. In the current study, the minimum values of the support and confidence were set as 5% and 65%, respectively, which were higher than those in similar studies (Fu et al., 2017).

It has been a consensus that the efficacy of TCM depends on multi-ingredients rather than a single ingredient. Therefore, identifying the hepatoprotective ingredients of specific TCM as complete as possible would help explain its hepatoprotection comprehensively. Generally, researchers compile the efficient ingredients of TCMs through surveying a great deal of literature. However, it is time-consuming and labor-intensive to conduct systematical literature retrieval. Therefore, an efficient and effective method to identify the hepatoprotective ingredients of specific TCM was significant to elucidate its hepatoprotection. In the present study, a “TCM-ingredient” network focused on hepatoprotection was constructed, making it possible to identify the hepatoprotective ingredients of specific TCM efficiently and comprehensively. However, we must acknowledge that there is still some space for improvement in the performance of our hepatoprotective “TCM-ingredient” network. After all, ginkgolide C, one of the hepatoprotective ingredients of Yin Xing Ye, was not successfully discovered. With the introduction of more TCMs and ingredients into the network, it would provide more reliable and complete results in future research.

Another interesting contribution of this work was that a material basis and drug property-based method were originally proposed to discover novel potential hepatoprotective TCMs. This method integrated information on the active ingredients and drug properties of TCMs comprehensively. Based on the method mentioned earlier, a total of 12 potential hepatoprotective TCMs were discovered. Both the efficacy category analysis and the association rules analysis supported the reliability of our results. In addition, 9 out of the 12 potential hepatoprotective TCMs were reported to relieve or treat various types of liver disorders, indicating the effectiveness of our method further. The hepatoprotective activities of Ling Xiao Hua, Gao Ben, and Gu Sui Bu were not reported. They were expected to be novel hepatoprotective drugs. Therefore, experimental verification focused on the hepatoprotective activities of these three TCMs was urgent and imperative in the next step of our research. One limitation to the method was that the contents of the hepatoprotective ingredients were not taken into consideration. We must acknowledge the importance of the dose-effect relationship. However, it was very difficult to collect the dose effect relationship information of so many hepatoprotective ingredients. When sufficient quantitative data are available, the quantitative methodology study would make great progress. In the future, based on the method proposed in the current study, we believe that more TCMs that were not reported to protect the liver previously will be gradually uncovered to show hepatoprotective effects.

## 4 Materials and methods

### 4.1 Construction of *in silico* models for predicting the hepatoprotective activity of phytoconstituents derived from traditional Chinese medicines

#### 4.1.1 Data sources

In our previous study, focusing on herbal-induced liver injury, we developed a dataset consisting of 664 hepatoprotective phytoconstituents and 216 hepatotoxic phytoconstituents (He et al., 2019b). This dataset laid the foundation for exploring the hepatic effects induced by phytoconstituents based on a data-driven method. Recently, we collected 13 novel hepatoprotective phytoconstituents. They were also added to the dataset of herbal-induced liver protection (Supplementary File S6). Here, the dataset mentioned earlier was utilized to develop a computational molecular model for evaluating the hepatoprotective activity of phytoconstituents. Of note, the phytoconstituents included in the dataset of herbal-induced liver protection were defined as positive samples, whereas the chemical ingredients included in the dataset of herbal-induced liver injury were regarded as negative samples. Both the positive and negative samples were divided into training and test sets in the ratio of 4 to 1 by implementing the Kennard–Stone algorithm. Finally, the training and test sets contained 709 (538 positives and 171 negatives) and 178 (135 positives and 43 negatives) diverse chemicals, respectively (Supplementary File S7).

#### 4.1.2 Molecular descriptor calculation and feature selection

PaDEL-Descriptor (version 2.2.1) (Yap, 2011), a powerful and freely available software application to calculate molecular descriptors and fingerprints, was applied to calculate the two-dimensional (2D) structures of the phytoconstituents. A total of 1444 2D descriptors were taken into consideration, including information on the physicochemical properties and topological geometry properties of the chemicals. With the aim of minimizing the redundancy of the feature variables, the Boruta algorithm (Kursa and Rudnicki, 2010) was implemented to identify feature variables associated with the outcome variable. Thereafter, Pearson’s correlation analysis was conducted to eliminate the highly correlated feature variables. The maximum threshold of the Pearson’s correlation coefficient was set at 0.90. Finally, a total of 80 non-redundant feature variables were retained and used as input to develop a computational molecular model (Supplementary File S7). Of note, there were six compounds that could not be recognized by PaDEL-Descriptor. Therefore, the total sample size of the training and test sets was 887 rather than 893.

#### 4.1.3 Model construction and evaluation

In the past decades, a series of machine learning algorithms have been proposed by mathematicians and statisticians. However, owing to each algorithm and its own merits, it was difficult to define which one was the best. With the aim of attaining a relatively optimal model, a total of eight algorithms were investigated, including Naive Bayes, J48, K-star, IBK, random forest, Bagging-IBK, AdaBoost-J48, and voting. The rationales of these eight algorithms mentioned earlier have been reviewed in our previous publication (He et al., 2019). In addition, CVParameterSelection, an effective parameter optimization method, was adopted to confirm the optimum parameters for each algorithm. All of the algorithms mentioned earlier were implemented *via* the Waikato Environment for Knowledge Analysis (WEKA, version 3.8.3) platform within 5-fold cross-validation (Frank et al., 2004).

Three indicators were used to evaluate the predictive ability of the models, including ACC, SE, and SP. These indicators stand for the predictive accuracy of the overall, positive, and negative samples, respectively. In addition, the AUC, an important index for measuring the model’s comprehensive performance, was also calculated (Linden, 2006).

### 4.2 Identification of representative substructures for the hepatoprotective activity

SARpy (version 1.0) is a tool to associate structural fragments with specific activity/toxicity (Ferrari et al., 2013). It has been proven to be effective and efficient to extract RSs in many previous studies. Here, it was utilized to identify RSs for hepatoprotection. The minimum thresholds were set at 10 for both the likelihood ratio (LR) and the frequency. The former and the latter indices represent the predictive power and the occurrence number of specific RS, respectively. For a given RS, the greater the LR value, the stronger is its predictive power.

### 4.3 Comprehensive analysis focused on the hepatoprotective activity of traditional Chinese medicines at a singular traditional Chinese medicine level based on bioinformatics

#### 4.3.1 Data sources

To collect hepatoprotective TCMs, CNKI (China National Knowledge Infrastructure) and PubMed databases were retrieved according to the procedure listed as follows. First, the publications related to TCMs were identified *via* key phrases of Traditional Chinese Medicine, TCM, herbal, herb, medicinal plant, and botanical. The time span was restricted to between 2012 and 2021. Thereafter, a series of search terms, including hepatotoxicity, liver toxicity, liver injury, liver damage, hepatitis, liver fibrosis, liver failure, liver cancer, hepatoma, liver tumor, liver neoplasms, hepatocellular carcinoma, liver cirrhosis, hepatomegaly, fatty liver, jaundice, cholestasis, liver protection, hepatoprotective, and hepatoprotection, were applied to screen the literature related to TCMs. Only those literature reports associated with herbal-induced hepatoprotection were retained. Finally, hepatoprotective TCMs were collected by reading the literature systematically. Of note, only those TCMs recorded in the Chinese Pharmacopoeia (2020 edition) were taken into consideration in the current study. The efficacy category and the drug property information of each TCM were extracted from TCMSP (Traditional Chinese Medicine Systems Pharmacology Database and Analysis Platform) (Ru et al., 2014) and the Chinese Pharmacopoeia (2020 Edition) (Chinese Pharmacopeia Commission, 2020), respectively.

#### 4.3.2 Statistical analysis

Statistical analysis was performed by SPSS version 17.0 (SPSS Inc., Chicago, United States). The data were presented by frequency. A two-tailed chi-squared test was used for comparing the drug properties (four properties, five flavors, and channel tropism) of the hepatoprotective and non-hepatoprotective TCMs. *p* < 0.05 was considered statistically significant.

#### 4.3.3 Association rules analysis

Association rules analysis was conducted by implementing the Apriori algorithm embedded in the WEKA platform (version 3.8.3) (Frank et al., 2004). The minimum values of support and confidence were set at 5% and 65%, respectively.

#### 4.3.4 Construction of the “traditional Chinese medicine-ingredient” network

Over the past decades, many TCM databases have been developed, providing us an opportunity to investigate the relationships between TCMs and chemical components. Here, a total of three typical TCM databases, including TCMSP (Ru et al., 2014), ETCM (Xu et al., 2019), and TCMID (Huang L. et al., 2018), were adopted. By taking the hepatoprotective ingredients collected in Section 2.1.1 as input, a series of “TCM-ingredient” relationship data were attained. Then, a comparison between the TCMs attained earlier and the hepatoprotective TCMs collected in Section 2.2.1 was conducted. According to whether or not the TCMs were reported to produce beneficial effects on the liver, the “TCM-ingredient” relationships were divided into two categories: hepatoprotective “TCM-ingredient” relationships and undetermined “TCM-ingredient” relationships. Undetermined means that whether the TCMs benefit to the liver or not was unknown. Finally, the former and the latter relationship data were utilized to construct the hepatoprotective “TCM-ingredient” network and an undetermined “TCM-ingredient” network, respectively. To realize the visualization of the network, Gephi (version 0.9.2) software was adopted (Jacomy et al., 2014).

#### 4.3.5 Hierarchical cluster analysis

Herein, taking drug properties of the hepatoprotective TCMs (123) and the non-hepatoprotective TCMs (132) as input, hierarchical cluster analysis based on the Euclidean distance was conducted to develop a cluster model. To perform the hierarchical cluster analysis, Origin 9.0 software was used. The drug properties were presented by a binary variable with values of 0 or 1, for example, for TCM Chuan Xiong (Chuanxiong Rhizoma), warm properties, pungent flavor, and entering the liver, gall bladder, and pericardium meridians. Its drug properties were presented as cold 0, hot 0, warm 1, cool 0, neutral 0, pungent 1, bitter 0, sweet 0, sour 0, salty 0, astringent 0, tasteless 0, liver 1, lung 0, stomach 0, spleen 0, kidney 0, heart 0, large intestine 0, gall bladder 1, bladder 0, small intestine 0, pericardium 1, and triple energizer 0.

## 5 Conclusion

In this work, a large-scale dataset of TCM-induced hepatoprotection was constructed through investigating a great deal of literature. Then, the dataset was analyzed based on the structure–activity relationship, molecular network, and machine learning techniques comprehensively. Finally, we developed predictive models for TCM-induced hepatoprotection at the level of molecule and singular TCM, respectively. The model at the molecule level held a strong predictive power with an accuracy greater than 85%. It would provide valuable clues for researchers to screen potential hepatoprotective ingredients from TCMs. The model at the singular TCM level innovatively integrated the material basis and drug property information. It would aid the discovery of novel potential hepatoprotective TCMs. Literature searches showed that the results produced by the singular TCM level model were highly consistent with the reports in previous publications. In summary, we developed effective and reliable predictive models for TCM-induced hepatoprotection at the level of molecule and singular TCM, respectively. Such comprehensive predictive research would be highly desirable for screening and discovering novel potential hepatoprotectants from TCMs. In addition, the research approaches used in the current study also provided a highlighted mode for discovering the novel functions of TCMs.

## Data Availability

The original contributions presented in the study are included in the article/Supplementary Material; further inquiries can be directed to the corresponding authors.
